# Wheat Roll Enhanced by Buckwheat Hull, a New Functional Food: Focus on the Retention of Bioactive Compounds

**DOI:** 10.3390/molecules28114565

**Published:** 2023-06-05

**Authors:** Małgorzata Wronkowska, Natalia Bączek, Joanna Honke, Joanna Topolska, Wiesław Wiczkowski, Henryk Zieliński

**Affiliations:** Department of Chemistry and Biodynamics of Food, Division of Food Science, Institute of Animal Reproduction and Food Research of Polish Academy of Sciences, Tuwima 10, 10-748 Olsztyn, Poland; m.wronkowska@pan.olsztyn.pl (M.W.); n.baczek@pan.olsztyn.pl (N.B.); j.honke@pan.olsztyn.pl (J.H.); j.topolska@pan.olsztyn.pl (J.T.); w.wiczkowski@pan.olsztyn.pl (W.W.)

**Keywords:** Maillard reaction products, antioxidant capacity, tocopherols, wheat roll, buckwheat hull

## Abstract

Wheat roll enhanced by buckwheat hull was used as a model for determining the retention of bioactive compounds during technological steps. The research included analysis of the formation of Maillard reaction products (MRPs) and retention of bioactive compounds such as tocopherols, glutathione, or antioxidant capacity. About a 30% decrease in the content of available lysine in the roll was observed compared to the value obtained for fermented dough. Free FIC, FAST index, and browning index were highest for the final products. The increase of analyzed tocopherols (α-, β-,γ-, and δ-T) was noticed during the technological steps, with the highest values found for the roll with 3% of buckwheat hull. A significant reduction in GSH and GSSG content occurred during the baking process. The observed increase in the value of the antioxidant capacity after the baking process may be the result of the formation of new antioxidant compounds.

## 1. Introduction

According to available statistical data, bread sale values are expected to grow annually by 5.87%, based on the compound annual growth rate in 2023–2027. The average volume per person in the “Bread segment” is expected to amount to 23.46 kg in 2023 [1]. Bread and bakery products are still basic products in the diet of most people in the world. However, consumers are looking for information regarding not only the nutritional value of baking products but also the ability to reduce some civilization diseases, for example, heart disease, diabetes, and cancer [2]. Such an ingredient may be, for example, the buckwheat hull obtained during the production process of kasha or flour, as proposed in this work. As shown in our previous study by Wronkowska et al. [3], buckwheat ingredients affect the sensory qualities of bread/rolls without impacting consumer acceptance, and also improve their microbial qualities. Further, from the literature data, we know that buckwheat hulls contain a high concentration of flavonoids [4], and they have the highest dietary fiber content, total polyphenol content, and antioxidant activity compared to whole or dehulled buckwheat seeds [5]. Čabarkapa et al. [6] showed the potential antimicrobial activity of buckwheat hull extracts against six species of Gram-positive and Gram-negative bacteria present in food.

During the thermal processing of food, as well as during its long-term storage, many chemical changes occur, initiated by a direct reaction between the reducing sugars and amino acids or peptides. This process is very complex and leads to the formation of compounds responsible for the taste, smell, and attractiveness of food products and is called the Maillard reaction (MR) [7]. The products of the reaction are both substances considered carcinogenic and mutagenic (e.g., acrylamide), as well as substances showing antioxidant and antibacterial properties (e.g., melanoidin), which can have a positive effect on the human body. However, during high-temperature processing, MR can also produce potentially toxic and harmful chemicals, advanced glycation end products (AGEs). AGE accumulation is associated with diabetes, inflammation, and diseases caused by aging [8]. In the human body, the formation of AGEs is a part of normal metabolism, but they can become pathogenic if their levels in tissue and circulation are extensively high [9]. In vivo, the formation of AGEs and intake of dietary AGEs may result in their excessive accumulation in the human body [10]. The search for glycation inhibitors is of interest, and a variety of synthetic and natural products have been evaluated as inhibitors of AGE formation [11]. In the review by Khan et al. [12], there are some examples of inhibition of AGEs by polyphenols such as the resveratrol and phytoestrogen from grapes or hydroxytyrosol (2-(3,4-hydroxyphenyl) found in the mill waste of olives. It should also be remembered that heat treatment during the roasting and baking process affects the formation of potentially harmful Maillard reaction compounds, such as acrylamide (ACR), hydroxymethylfurfural (HMF) furfural, and especially advanced glycation end products (AGE), e.g., Nε-(carboxymethyl)lysine) [13].

Previous research, also regarding buckwheat hulls, has suggested that dietary polyphenols, depending on the chemical structure, may serve as effective AGE inhibitors and contribute to the prevention of processes resulting in aging and diabetes complications [14,15,16,17]. Other scientists also present the possibility of using buckwheat hulls as a valuable ingredient in food products. Sujka et al. [18] found that it could be used as an ingredient in wheat pasta because of its high phenolic content and antioxidant activity. However, due to the sensory quality, the level of buckwheat hull addition to the pasta should be no more than 10%. In addition, Liu et al. [19] showed that the addition of no more than 4% of buckwheat hull did not decrease the wheat dough properties and noodle quality. Hęś et al. [20] used buckwheat hull extract in meat products, and they found that this ingredient could be used to prolong the shelf life of frozen meat products by protecting them against lipid oxidation and deterioration of their nutritional quality.

In our research, we proposed the use of buckwheat hull as an additive to one of the popular and available baking products (wheat roll) in the Warmia and Mazury regions of northern Poland. From our previous research, we know that this product has been approved by consumers [3]. Therefore, in this study, the aim was the determination of how the technological cycle used to obtain the final product, which is wheat roll enhanced by buckwheat hull, affects the formation of Maillard reaction products (MRPs) and retention of bioactive compounds, tocopherols, or glutathione.

## 2. Results and Discussion

Data presented in Table 1 show the formation of Maillard reaction products (MRPs), selected bioactive compounds, the antioxidant capacity, and the ability to inhibit the formation of AGEs in two model systems as an effect of technological steps producing buckwheat hull-enhanced roll. The dough production process as well as its fermentation did not significantly increase the content of available lysine during preparation of the roll with 3% of buckwheat hull. However, the baking process decreased its content significantly, by about 30% compared to values obtained for fermented dough. Lysine (from 0.8 g to 3.7 g 100 g^−1^ protein) and also essential amino acids (EAAs) such as methionine, threonine, and tryptophan occur in very small amounts in cereal crops [21]. Therefore, in a diet rich in grains, often the first limiting amino acid is lysine, and the recommended daily dietary intake of lysine is 40–180 mg kg^−1^ d^−1^ [22]. Mustafa et al. [23] analyzed amino acids in different fractions and products of wheat, rye, oats, and barely. They found that the bran fraction of all analyzed cereals contained more free amino acids (also lysine) than whole grain or sifted flour. Fermentation and baking seem to expend, as well as release, certain amino acids [23]. In the case of wheat, these authors found an increase in lysine content after dough fermentation and baking. The heat treatment of products containing proteins and reducing sugars results in the formation of Maillard reactions, and a protein–carbohydrate complex is formed. This complex contains an unreactive (unavailable) lysine, which is bound to reducing sugars and is not available in the body, so the nutritive value of foods decreases [24]. Brestenský et al. [25] showed that the amino acid could play an important role in the final antiradical activity of the compounds, and previous assays showed the ability of lysine to scavenge free radicals. Therefore, the disappearance of free lysine with increased heating time could have contributed to the reduced antiradical properties of the samples. Yang et al. [26] reviewed the lysine metabolic pathway—this is one of the most important for human health, maintaining immunity, building structural proteins for connective tissues, and controlling calcium homeostasis and fatty acid metabolism. In bakery products, the amount of the antioxidant and chemopreventive compound pronyl-L-lysine is strongly dependent on the recipe and technological processes used, as presented by Lindenmeier and Hofmann [27]. These authors also found a higher amount of pronyl-L-lysine in sourdough bread compared to yeast-fermented bread.

The fluorescence of free intermediary compounds (FIC) formed at the advanced MR stage was analyzed. The FAST index, the ratio of FIC to soluble tryptophan, indicates the loss of nutritional quality due to the extent of the Maillard reaction at advanced steps in food production. Generally, the increase of both indicators was observed, compared with the mixture of flours (F). The highest values of FIC and FAST index were noticed for the final product, buckwheat hull-enhanced wheat roll (Table 1). Bhinder et al. [28] showed that for eight Tartary buckwheat varieties, infrared roasting increased the FAST index, and FIC and roasting temperature showed a highly significant positive correlation. A review by Liogier de Sereys et al. [29] showed the potential of the FAST index to characterize infant formula quality. These authors presented this indicator as simple measurements which allow rapid characterization of infant formula quality at different steps of the process, from raw dairy material to final product, and it could be used as a rapid tool for quality control.

**Figure 1:**
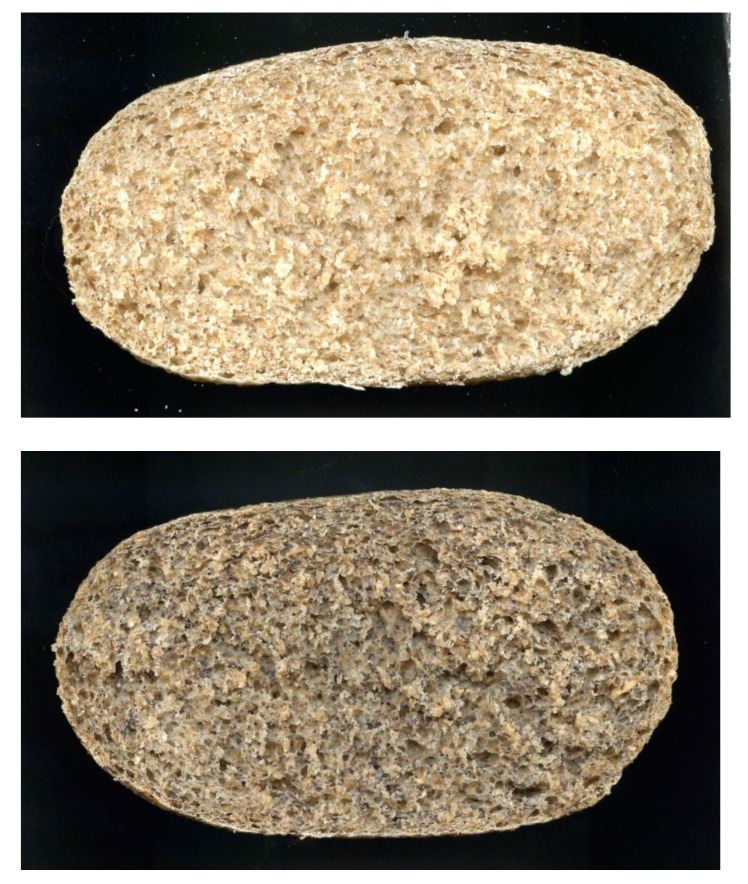
Wheat roll: control (**top**) and with the addition of 3% of raw buckwheat hull (**bottom**).

In the final stage of the Maillard reaction, one of the products formed is melanoidin, and the indicator of its formation is the determination of the browning index (Figure 1). Dough preparation and fermentation processes affect the increase in the browning index value (Table 1). During the fermentation process, some proteins and reducing sugars could be released from the dough matrix, and it could be related to the increase of the browning index noticed compared to the flour mixture. The most important changes were observed during baking—about a 40% increase in the browning index value was noticed compared to sample F. Phenolic compounds are involved in melanoidin formation and provide melanoidin with antioxidant activity, as was shown by Brudzynski and Miotto [30]. These authors showed that melanoidin formation in honey was increased by heat treatment and was associated with both enhanced incorporation of phenolics into the protein-based melanoidin skeleton and an increase in polyphenol–protein complexation.

Tocopherols and tocotrienols are a group of naturally occurring antioxidants commonly referred to as vitamin E (α-T, β + γ-T, and δ-T) and δ-tocochromanols [31]. Compared with a mix of flour used for baking (F), all analyzed tocopherols (α-, β-,γ-, and δ-T) increased during the technological steps, and their highest content was found in the final product (R) (Table 1). The β-,γ-, and δ-tocochromanols are not converted to α-tocopherol in humans, so α-tocopherol alone should be used for estimating requirements and recommendations for vitamin E intake [32]. Maruyama et al. [33] found only α-tocopherol in white bread enriched with chia seeds and carrot leaves. This fact can be justified because the free radical scavenging activity of tocopherols is highest in δ-tocopherol followed by γ-, β-, and α-tocopherol. Damanik and Murkovic [34] showed that tocotrienols are more stable than tocopherols when exposed to heat treatments. Wholegrain buckwheat flour was the richest source of vitamin E, and especially of the γ-tocopherol, compared to wheat flour, as presented by Sedej et al. [35].

In the mixture of flours for baking of a roll enriched with buckwheat hull (F), the content of reduced glutathione (GSH) was 386.14 nmol/g dm and oxidized glutathione (GSSG) 162.63 nmol/g dm. In pre-fermented dough, these contents increased slightly, by 12.9% in the case of GSH and 31.5% in the case of GSSG. The dough fermentation process reduced the GSH and GSSG levels by 4% and 9.3%, respectively. Significant reduction in GSH and GSSG content occurred during the baking process. The amount of reduced glutathione decreased by 74% and the amount of oxidized glutathione by 37.7%. GSH, apart from the phenolic compounds, is known to be an important antioxidant and anticarcinogen and might be important for protecting against damage from free radicals [36]. Dough rheology and breadmaking performance depend on the polymeric structure of the glutenin protein fraction. In flour, the tripeptide glutathione (7-glutamylcysteinylglycine) exists in two forms: the free reduced (GSH) and free oxidized (GSSG), as well as in the form of protein-glutathione mixed disulfides (PSSG). Glutathione is involved in the redox reactions that occur in dough [37]. In freshly milled wheat flour, the GSH content was 80.7 nmol/g, and for GSSG, it was 16.9 nmol/g, as presented by Chen and Schofield [38].

Analyzing the obtained results, it was found that the technological processes used during the production of roll enriched by buckwheat hull had a significant impact on the changes in antioxidant capacity of intermediate products, i.e., unfermented and fermented doughs, and obtained bakery products (Table 1). The dough preparation process increased the value of the antioxidant capacity determined against the ABTS^•+^, DPPH^•^, and superoxide anion (O_2_^−•^) radical in all tested nonfermented dough samples (D) compared to the flour mixture (F). The same observations were made for the other analyzed samples: dough after fermentation and the final bakery product. The observed increase in the value of the antioxidant capacity after the baking process may be the result of the formation of new antioxidant compounds. These compounds are mainly high-molecular-weight melanoidin, which forms as a result of the Maillard reaction occurring at a high temperature between the free amino group of proteins, peptides, or amino acids and the residues of reducing sugars [39].

The fermentation process used significantly increases antioxidant activity. As was shown in the review by Zhao et al. [40], fermentation involves a series of reactions that modify the chemical components of the substrate. These authors indicate that during fermentation, various factors affect the production of bioactive components. They include, among others, starting microorganisms, pH value, fermentation time, fermentation type, and enzymes produced during this process. Dapčević-Hadnađev et al. [41] found that wheat variety, type of fermentation, and processing affected antioxidant activity. They showed in their research, as in our study, that antioxidant activity increased after sourdough fermentation and slightly decreased or remained the same after baking. However, in their study, the reduction of antioxidant capacity was noticed after dough mixing, whereas in our research, the increase of ABTS, DDPH, and PCL values was found.

**Figure 2:**
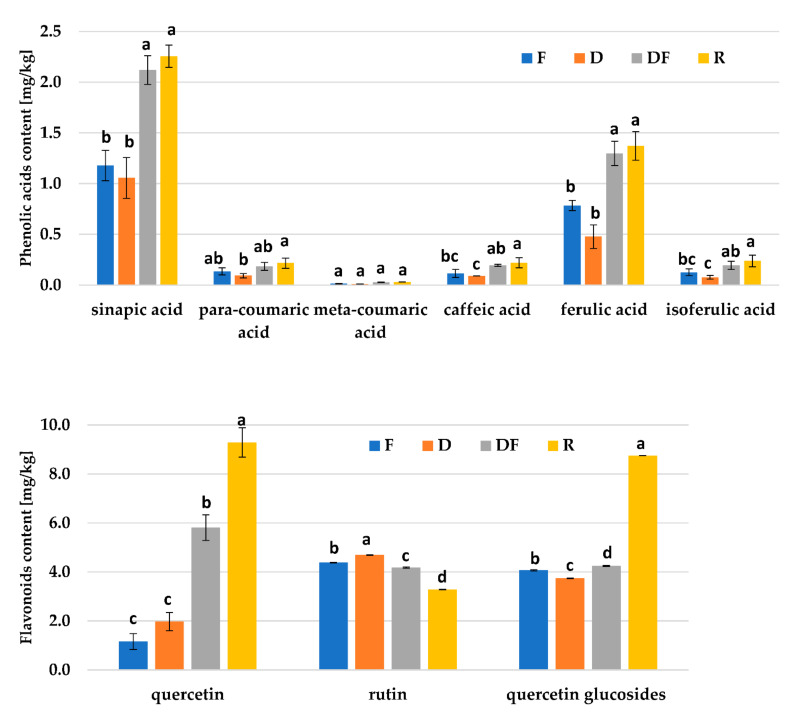
The content of phenolic acids (**top**) and flavonoids (**bottom**) in the technological cycle. Values with different letters are significantly different (*p* < 0.05). F—a mixture of wheat flour and buckwheat hull; D—dough before fermentation; DF—dough after fermentation; R—roll with 3% of buckwheat hull.

Nine substances from the group of phenolic acids and flavonoid compounds were found in the analyzed samples in the technological cycle from the flour mix to the final product (Figure 2). Based on the analyses carried out, the presence of para-coumaric acid, meta-coumaric acid, sinapic acid, ferulic acid, isoferulic acid, caffeic acid, rutin, quercetin, and quercetin glucosides was found. In addition, it was shown that the identified phenolic acids and flavonoid compounds were present in the analyzed samples in free, ester, and glycoside forms. The obtained results indicate that the technological processes used during preparation of wheat roll enhanced with buckwheat hull caused an increase in the content of phenolic acids, which was probably due to the release of these substances from the cell wall structures as a result of the applied mechanical (dough mixing), fermentation, and thermal processes (baking). Similar observations were made by Yu and Beta [42] during the production process of bread from purple wheat grains, and Tian et al. [43] during the bread-making processes using four different varieties of hard red winter wheat. The degradation of the buckwheat grain cell membrane during heat treatment could enhance the extractability of phenolic compounds from the bound fraction [44]. The main phenolic acids found in the investigated material were sinapic (from 1.18 to 2.26 mg/kg) and ferulic acids (from 0.78 to 1.37 mg/kg). A decrease in the level of rutin and an increase in the content of quercetin was observed as a result of the processes used in the technological cycle. The applied thermal process and fermentation resulted in the release of quercetin from its rhamnoglucoside form. Sedej et al. [35] found a significantly higher content of phenolics in buckwheat than in wheat flour, which suggested the possibility of improving the antioxidant properties of wheat-based food products through the addition of buckwheat products. Tian et al. [43] showed that the bread-making process generally had a positive effect on the phenolic profile and antioxidant activities of wheat products. They found that the fermentation process generally increased soluble phenolic content, flavonoid content, and antioxidant activities, while the baking process increased the soluble phenolic content and antioxidant activities. In addition, these authors showed that some phenolic acids were incorporated into Maillard reaction products during baking.

The phenolic acids were positively correlated with analyzed parameters: FIC (r = 0.80, *p* < 0.05), browning index (r = 0.70, *p* < 0.05), and antioxidant capacity (r = 0.72, *p* < 0.05). However, a significantly higher correlation coefficient for flavonoids and analyzed parameters was noticed: FIC (r = 0.99, *p* < 0.05), browning index (r = 0.94, *p* < 0.05), and antioxidant capacity (r = 0.68, *p* < 0.05). This may suggest that the MRPs and antioxidant properties observed in the analyzed samples are related to the flavonoids present rather than to the phenolic acids. In a review, Teng et al. [45] presented the impact and inhibitory mechanism of phenolic compounds on the formation of toxic MRPs in food, such as acrylamide, heterocyclic amines, and AGEs. Billaud et al. [46] reported that MRPs can significantly inhibit the initial step of enzymatic browning caused by polyphenol oxidase and tyrosinasesmediated oxidative degradation of phenolic compounds produced in fruits and vegetables.

## 3. Materials and Methods

### 3.1. Materials

Commercially available wheat flour types 750 and 1850 and the ground hull obtained during the milling of common buckwheat were used in this research. The study included the following materials: a mixture of wheat flour and buckwheat hull used for obtaining a roll (F), dough before fermentation (D), dough after fermentation (DF), and roll with 3% of buckwheat hull (R). The buckwheat hull additive used was selected based on our previous work [3]. The roll with 3% of buckwheat hull was obtained as follows: the wheat flour types 750 (14%) and 1850 (40%) were mixed with buckwheat hull (3%), and salt (1%), sugar (2%), and yeast (2%) were dissolved in water (38%). After dough fermentation (for 60 min), it was divided into rolls and fermented again for 30–40 min at 30 °C, at a humidity of 75–80%. Rolls were baked in traditional ovens for about 20 min at 260 °C. Buckwheat hull-enriched rolls were produced in the small bakery located in Szczytno, from the region of Warmia and Mazury, Poland.

### 3.2. Methods

Assays of available lysine, free fluorescence intermediary compounds (FIC), FAST index (fluorescence of advanced Maillard reaction products and soluble tryptophan), and browning index were conducted according to methods described in detail by Michalska et al. [47]. Filtrates used for these analyses were obtained after incubation of dry samples with 6% SDS (30 min with stirring). The content of available lysine was determined by using the OPA assay (fluorescence at λ_Ex_ = 340 and λ_Em_ = 455 nm).

The free fluorescence of intermediary compounds (FIC) was measured at λ_Ex_ = 353 and λ_Em_ = 438 nm. Analysis of fluorescence due to advanced MRPs (λ_Ex_ = 353 and λ_Em_ = 438 nm) and tryptophan fluorescence (λ_Ex_ = 290 and λ_Em_ = 340 nm) was used for the FAST index. For the formation of brown pigments, the investigated samples were extracted with phosphate buffer (60 min at room temperature, pH 7.4), and then after filtration, the assay was performed at the absorbances 420 and 360 nm (Coulter DU 800 spectrophotometer, Beckman Instruments Inc., Fullerton, CA, USA).

A modified method according to Zieliński et al. [48] was used for tocopherol (δ-T, α-T, β-T, γ-T) analysis. Samples were extracted with methanol (0.3 g of sample/1 mL), evaporated, and extracts were redissolved in acetonitrile. The tocopherols were separated by HPLC UltiMate 3000 Dionex equipped with a fluorescent detector RS (column C18 Kinetex Phenomenex 100A).

Determination of reduced (GSH) and oxidized glutathione (GSSG) was made according to Zieliński et al. [49]. Three grams of samples were mixed with phosphate buffer and potassium chloride and homogenized. Then, polyvinylpolypyrrolidone (PVPP) was added, and after thorough mixing, the mixture was centrifuged. The supernatant was kept on ice and assayed for reduced and oxidized glutathione at λ = 350 nm or 420 nm using a Perkin-Elmer LS 50 B Luminescence Spectrometer (Perkin-Elmer Ltd., Beaconsfield, Buckinghamshire, England).

For antioxidant capacity analysis, the determination against ABTS^+∙^ radical cation and PCL assays were used [17,49]. Samples (100 mg) were extracted with 80% methanol, and the supernatant obtained after repeated steps (extraction and centrifugation) was collected. Extract from analyzed samples was determined against ABTS^+∙^ radical cation using a spectrophotometric assay. Photochemiluminescence (PCL) assay with PHOTOCHEM^®^ apparatus (Analytik Jena, Leipzig, Germany) was used to measure the antioxidant capacity of the samples against superoxide anion radicals (O_2_^−•^). The total antioxidant capacity (PCL) was calculated as the sum of the values obtained for lipophilic (ACL) and hydrophilic (ACW) extracts of samples.

For the extraction and isolation of the main phenolic compounds, extraction with 80% methanol from analyzed samples was performed according to Wiczkowski et al. [50]. Free forms of polyphenols were isolated by diethyl ether, but conjugated form (esters and glycosides) samples were initially hydrolyzed with NaOH and HCl. After each hydrolysis, the extraction process was conducted in triplicate, and the collected ether extracts were evaporated to dryness. For analysis of the profile and content of phenolic acids and flavonoids, the system HPLC-MS/MS involving a HALO column was applied [50].

### 3.3. Statistical Analysis

Data were presented as the mean results of three replications for each sample with the standard deviation. To determine the differences, one-way ANOVA (*p* < 0.05) was performed, and then for post hoc comparison, the Fisher LSD test was used, and Pearson correlations were also determined (STATISTICA for Windows, StatSoft Inc., Tulsa, OK, USA, 2001).

## 4. Conclusions

Retention of bioactive compounds during baking technological steps was performed on a model of wheat roll enhanced by 3% of buckwheat hull. In general, an increase in all analyzed parameters was found after the first stage, which was the dough formation process. On the other hand, the yeast fermentation process increases the level of tocopherols (expressed as the sum of the analyzed fractions) and antioxidant activity. A significant reduction in GSH and GSSG content occurred after the baking process. Antioxidant capacity after the baking process was comparable with the data obtained for dough after fermentation, and even the formation of neo-formed antioxidant compounds, such as melanoidins, was found.

## Figures and Tables

**Table 1:** Effect of technological steps producing buckwheat hull-enhanced roll: formation of Maillard reaction products (MRPs), selected bioactive compounds, and antioxidant capacity.

	F	D	DF	R
MRPs				
available lysine [mg/g DM]	3.13 ± 0.25 ^a^	3.19 ± 0.03 ^a^	3.37 ± 0.14 ^a^	2.27 ± 0.02 ^b^
intermediate MRPs:				
FIC [FI/mg DM]	270.68 ± 23.2 ^c^	296.41 ± 14.5 ^b^	317.76 ± 8.0 ^b^	383.79 ± 15.5 ^a^
FAST index [%]	40.92 ± 3.8 ^c^	49.51 ± 2.0 ^b^	34.28 ± 2.1 ^d^	108.80 ± 4.7 ^a^
final MRPs:				
browning index [AU]	0.419 ± 0.010 ^c^	0.484 ± 0.025 ^b^	0.491 ± 0.005 ^b^	0.586 ± 0.003 ^a^
Tocopherols [μg/g DM]				
α-T	4.18 ± 0.8 ^d^	11.25 ± 2.6 ^c^	38.86 ± 3.5 ^b^	268.13 ± 6.9 ^a^
β + γ-T	48.33 ± 3.8 ^c^	60.03 ± 2.1 ^b^	69.67 ± 1.6 ^b^	280.61 ± 7.8 ^a^
δ-T	8.58 ± 1.7 ^b^	10.44 ± 2.6 ^a^	7.99 ± 2.4 ^b^	12.53 ± 1.3 ^a^
sum of T	61.10	81.72	116.51	561.27
Glutathione [nmol/g DM]				
GSH	386.14 ± 7.57 ^c^	435.84 ± 4.51 ^a^	418.72 ± 2.93 ^b^	109.34 ± 0.36 ^d^
GSSG	162.63 ± 5.85 ^c^	213.88 ± 4.75 ^a^	193.94 ± 4.42 ^b^	120.92 ± 1.90 ^d^
GSH/GSSG	2.4	2.0	2.2	0.9
Antioxidant capacity [μmol Trolox/g DM]				
ABTS	2.52 ± 0.09 ^c^	3.01 ± 0.07 ^b^	3.37 ± 0.14 ^a^	3.27 ± 0.12 ^a^
DPPH	1.93 ± 0.04 ^d^	2.21 ± 0.04 ^c^	2.36 ± 0.04 ^a^	2.27 ± 0.02 ^b^
PCL:				
ACW	0.79 ± 0.01 ^c^	0.83 ± 0.01 ^b^	0.91 ± 0.00 ^a^	0.94 ± 0.02 ^a^
ACL	2.00 ± 0.03 ^b^	2.23 ± 0.08 ^a^	2.19 ± 0.03 ^a^	2.14 ± 0.03 ^a^
sum	2.79	3.06	3.10	3.07

Values are mean ± standard deviation (*n* = 3). Values in each row with different small superscript letters are significantly different (*p* < 0.05). F—a mixture of wheat flour and buckwheat hull; D—dough before fermentation; DF—dough after fermentation; R—roll with 3% of buckwheat hull; FI—fluorescence intensity; AU—arbitrary units; reduced (GSH) and oxidized glutathione (GSSG).

## Data Availability

The data presented in this study are included in the article.
